# Nonlinear Vibration Control Experimental System Design of a Flexible Arm Using Interactive Actuations from Shape Memory Alloy

**DOI:** 10.3390/s23031133

**Published:** 2023-01-18

**Authors:** Ximei Li, Guang Jin, Mingcong Deng

**Affiliations:** The Graduate School of Engineering, Tokyo University of Agriculture and Technology, Tokyo 183-8538, Japan

**Keywords:** interactive actuation, operator theory, nonlinear vibration control, robust right coprime factorization, shape memory alloy

## Abstract

The flexible arm easily vibrates due to its thin structural characteristics, which affect the operation accuracy, so reducing the vibration of the flexible arm is a significant issue. Smart materials are very widely used in the research topic of vibration suppression. Considering the hysteresis characteristic of the smart materials, based on previous simulation research, this paper proposes an experimental system design of nonlinear vibration control by using the interactive actuation from shape memory alloy (SMA) for a flexible arm. The experiment system was an interactive actuator–sensor–controller combination. The vibration suppression strategy was integrated with an operator-based vibration controller, a designed integral compensator and the designed *n*-times feedback loop. In detail, a nonlinear vibration controller based on operator theory was designed to guarantee the robust stability of the flexible arm. An integral compensator based on an estimation mechanism was designed to optimally reduce the displacement of the flexible arm. Obtaining the desired tracking performance of the flexible arm was a further step, by increasing the *n*-times feedback loop. From the three experimental cases, when the vibration controller was integrated with the designed integral compensator, the vibration displacement of the flexible arm was much reduced compared to that without the integral compensator. Increasing the number of *n*-times feedback loops improves the tracking performance. The desired vibration control performance can be satisfied when n tends to infinity. The conventional PD controller stabilizes the vibration displacement after the 7th vibration waveform, while the vibration displacement approaches zero after the 4th vibration waveform using the proposed vibration control method, which is proved to be faster and more effective in controlling the flexible arm’s vibration. The experimental cases verify the effectiveness of the proposed interactive actuation vibration control approach. It is observed from the experimental results that the vibration displacement of the flexible arm becomes almost zero within less time and with lower input power, compared with a traditional controller.

## 1. Introduction

Flexible structures are used in a wide variety of applications, for example, robotics [1,2], multiple joint human arms [3], gantry crane systems [4], aircraft vertical tails [5], various kinds of manipulators [6] etc. Flexible structures inevitably lead to vibration problems. In view of the vibration problems caused by flexible structures, much research on vibration suppression has been undertaken over the years. Reference [7] used a vibration-based damage detection method using structural health monitoring techniques. Reference [8] researched vibration reduction performance based on sliding mode control for vehicle seats. Reference [9] discussed an input shaping control technique to reduce vibrations in machining. Reference [10] considered smart intelligent sensors/actuators from piezoelectric (PZT) and poly vinyl derylene fluoride (PVDF) materials using PID techniques to control the vibrations for flexible aluminum mechanical cantilever beams. Reference [11] conducted a comparative study on the vibration characteristics of a flexible GFRP composite beam attached to an SMA, finding that SMA actuators are more effective than PZT actuators.

The development of smart materials has been reported for many vibration control applications, such as interactive actuators, shape memory alloys (SMA) [12,13] controlled by operator-based design [14], piezoelectric materials in the form of PZT actuators [15], piezoelectric sensors [16,17], and so on. Shape memory alloys (SMA) as interactive actuators have proven to be particularly beneficial in comparison to other actuation technologies when incorporated in applications demanding strict adherence to a set of compatibility (e.g., mechanical, biological, weight) and environmental limitations. Their use in miniaturised components, lightweight systems, sensing-actuating systems, low-noise or low-impact appliances, and self-sensing controllable devices is particularly noteworthy. The main characteristics expected of SMA actuators for these fields are portability and being lightweight [18]. From the perspective of physical structure, the interactive SMA actuators are used by the way of two very slim wires fixed to the double sides of the flexible arm, the materials of which are made of a shape memory alloy [13]. SMA embodies a unique thermomechanical phenomenon, and can be restored to its original shape or configuration by heating above a characteristic temperature when plastically deformed in the low-temperature martensitic phase. Shape memory effect (SME) is the thermally triggered shape recovery of SMA. When an electric current signal passes through the SMA wire, the heat generated by the Joule effect causes the SMA wire to generate an actuation force, that is, the strain of the bilateral SMA wires applies the bending moment to the flexible arm and the bilateral SMA wires interact to control the vibration of the flexible arm. The unique properties of SMA make it suitable for a wide range of applications. Reference [19] reviewed SMAs in robotics applications in terms of control aspects. Reference [20] presented an experimental investigation of mechanical vibration attenuation with superelastic SMA bending springs. Reference [21] designed SMA spring-based actuators with a combined micro-macro mechanics analytical approach and presented its experimental validation. Reference [22] described a soft haptic glove actuated with SMA. Reference [23] studied SMA-based smart wearables paired with robotic applications for human–robot interaction. The hysteresis phenomenon of interactive actuation from smart materials is likely due to the elastic property of materials with a lag when a force or field is removed; the subsequent hysteresis effects are experienced with a certain delay in time, and this could cause undesirable oscillations leading to the degradation of the tracking performance and even instability if neglected [24]. The PI model can describe the infinite-dimensional objects for the hysteresis nonlinearities. When using smart material as interactive actuators, hysteresis nonlinearity is one of the most important properties, which usually results in undesirable oscillations and instability. The existing hysteresis models include the Preisach model, the Prandtl–Ishlinskii (PI) model [25,26], the Duhem model and the Bouc–Wen model.

Some vibration control methods for flexible structures have been investigated in the literature. Wang, A. and Deng, M. [27] presented multivariable tracking control for a manipulator with uncertainties using robust right coprime factorization. References [28,29] considered the tracking performance of an *n*-times feedback loop and a double-sided SMA actuator for the flexible arm. Araújo, J.M. et al. [30] studied a two-link flexible robot arm’s vibration control with the effect of time delay. Hu, Y. and Ng, A. [31] developed a vibration approach integrated with a piezoelectric actuator for the circular plate. Considering the system with hysteresis nonlinearity and uncertainty, the stability of the nonlinear control system should be ensured. In addition, the desired vibration control performance should be guaranteed. Some studies have considered the control method for nonlinear systems, such as reference adaptive control [32], the sliding mode method [33], predictive control [34] and the Lyapunov method [35]. In previous research, an operator-based robust nonlinear system was used to control vibration to deal with sudden perturbations [36], as well as using uncertain non-symmetric backlash [37], and the Prandtl–Ishlinskii hysteresis model [38]. The robust stabilization condition and tracking performance of nonlinear feedback control systems were obtained by using robust right coprime factorization in [39].

In the previous studies, vibration control was used as a single-sided SMA actuator. An operator-based vibration control system using an SMA actuator was designed for a flexible arm in [13]. A flexible arm attached load’s vibration issue is discussed in terms of single-sided SMA actuation in [24]. The single-sided actuator has a larger input power for vibration control and a slow response time. Subsequently, a double-sided SMA actuator was applied to operator-based vibration control with an *n*-times feedback loop for improving the tracking performance in [28]. Moreover, the interactive SMA actuation was used to control the vibration displacement of a flexible arm with simulation results [29].

The main contribution of this work is a novel approach using interactive SMA actuation to deal with nonlinear vibration attenuation and the effect of hysteresis nonlinearity. In comparison with previous research, this paper proposes the concept of interactive SMA actuation to control the vibration of a flexible arm. The nonlinear vibration control experimental system design can be implemented using interactive SMA actuation. The proposed vibration control method achieved robust stability and tracking performance of the system, and the experimental results verified the effectiveness of the vibration control of the proposed method compared to the PD controller. The experimental cases examine the vibration controller with/without the designed integral compensator, the tracking performance of the *n*-times feedback loop, and the comparison of a PD controller. The effectiveness of the proposed vibration control method was validated by the experimental results. We outline the main sections that contain the problem setup in Section 2, the proposed interactive actuation vibration control strategy in Section 3, the results and discussion in Section 4, and the conclusion in Section 5.

## 2. Preliminary and Problem Setup

In this section, the experimental device is described. The flexible arm’s mathematical model was derived by Euler–Bernoulli beam theory, the interactive SMA actuator with the effect of the hysteresis property was formulated using the thermal model and the PI hysteresis model. The problem of the flexible arm’s nonlinear vibration control was motivated by an interactive smart actuation.

### 2.1. Experimental Setup

**Figure 1:**
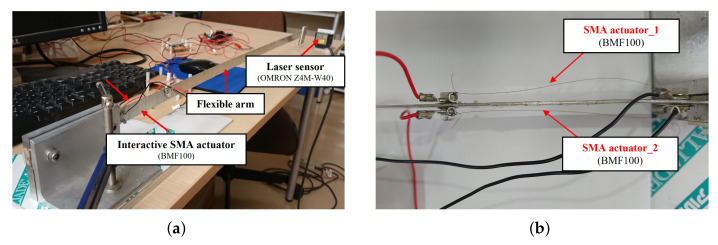
Configuration of the experimental setup. (**a**) The flexible arm. (**b**) Interactive SMA actuation.

**Figure 2:**
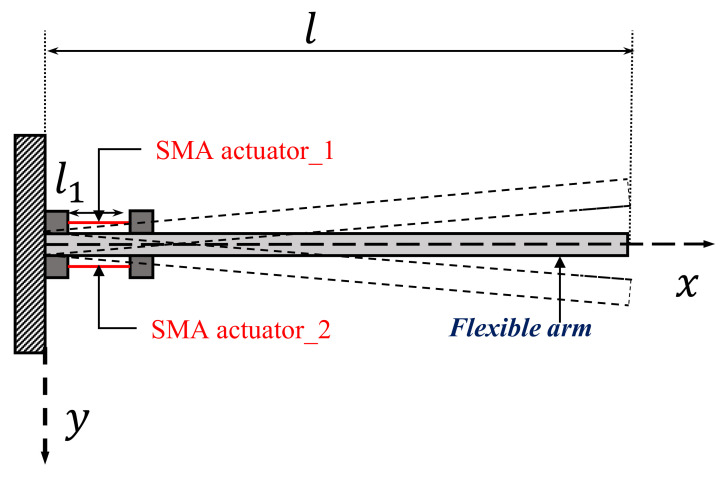
Schematic diagram of the experimental setup.

This experimental setup involves an interactive actuator–laser sensor–controller combination. The flexible arm was clamped to a rigid base at one end and was free at the other end. The double-sided SMA wire was attached to the flexible arm horizontally, and the deflection of the flexible arm was measured by a laser sensor fixed at the free end. In this experiment, the SMA wire was a BMF100 from Toki Corporation, the laser sensor was an Omron Z4W-W40. The vibration displacement was measured using the laser sensor, and the control input force was calculated on the PC via I/O (PCI-3522A) via A/D conversion, and then D/A conversion was stamped onto the SMA via the amplifier. In Figure 1, the overview of the experimental device is shown; Figure 1a shows the flexible arm and Figure 1b the interactive SMA actuator. The corresponding schematic diagram of the experimental device is represented in Figure 2. The physical parameters of the flexible arm are listed in Table 1.

### 2.2. Interactive SMA Actuator’s Thermal Model

**Figure 3:**
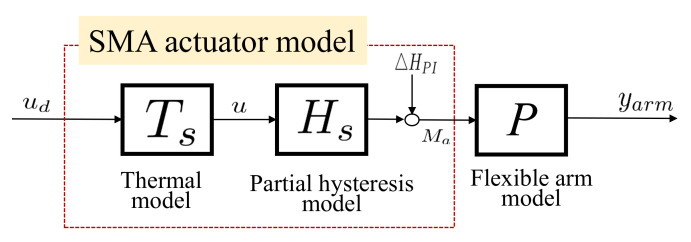
Interactive SMA actuator model.

The interactive SMA actuator’s thermal model is the relationship between temperature and electric power denoted as $T_{s}$. The illustration of the plant with the interactive SMA actuator is shown in Figure 3. The thermal model of the interactive SMA actuator is expressed by the heat conduction Equation (1) as follows:

(1) $$mc_{p}\frac{d(u\left(t\right)-T_{a})}{dt}=i^{2}\left(t\right)R-h_{c}A_{c}\left(u\left(t\right)-T_{a}\right),$$

where the ambient temperature $T_{a}$ is set to 20 degrees; the other parameters of the thermal model are listed in Table 1.

For convenience, the electric power $i^{2}\left(t\right)R$ is defined as the input signal $u_{d}\left(t\right)$ of the thermal model as in Equation (2),

(2) $$u_{d}\left(t\right)=i^{2}\left(t\right)R.$$

Then, Equation (1) can be transformed following Equation (3),

(3) $$u(t)=T_{s}\left(u_{d}\right)\left(t\right)=\frac{1}{mc_{p}}\int_{0}^{t}e^{-\gamma (t-\tau )}u_{d}\left(\tau \right)d\tau +T_{a}$$

(4) $$\gamma =\frac{h_{c}A_{c}}{mc_{p}}.$$

### 2.3. Interactive SMA Actuator’s Hysteresis Model

The mathematical model of PI is a superposition of play or stop operations which are chosen to describe hysteretic nonlinearities. In this paper, the PI hysteresis model refers to Reference [38]. The hysteresis operator can be expressed as Equation (5).

(5) $$F_{h}\left(u\right)\left(t\right)=\left\{\begin{array}{cc} u(t)+h, & \mathrm{if}u\left(t\right)\leq F_{h}\left(u\right)\left(t_{i}\right)-h\\ F_{h}\left(u\right)\left(t_{i}\right), & \mathrm{if}-h<u\left(t\right)-F_{h}\left(u\right)\left(t_{i}\right)<h\\ u(t)-h, & \mathrm{if}u\left(t\right)\geq F_{h}\left(u\right)\left(t_{i}\right)+h. \end{array}\right.$$

*p*(*h*) is a given continuous density function.

(6) $$p\left(h\right)=a\times e^{b{\left(h-1\right)}^{2}}.$$

Then, the PI model can be redescribed by a weighted superposition of the above play hysteresis operator as follows:

(7) $$u^{*}\left(t\right)=\int_{0}^{H}p\left(h\right)F_{h}\left(u\right)\left(t\right)dh=H_{s}\left(u\right)\left(t\right)+\Delta H_{PI}\left(u\right)\left(t\right),$$

where the two operators of $H_{s}$ and $\Delta H_{PI}$ are denoted as Equations (8) and (9), respectively.

(8) $$H_{s}\left(u\right)\left(t\right)=K\cdot u\left(t\right),K=\int_{0}^{h_{x}}p\left(h\right)dh$$

(9) $$\Delta H_{PI}\left(u\right)\left(t\right)=-\int_{0}^{h_{x}}S_{0}hp\left(h\right)dh+\int_{h_{x}}^{H}p\left(h\right)F_{h}\left[u\right]\left(t_{i}\right)dh.$$

(10) $$S_{0}=\left\{\begin{array}{cc} 1, & \mathrm{if}u\left(t\right)-F_{h}\left[u\right]\left(t_{i}\right)\geq 0\\ -1, & \mathrm{if}u\left(t\right)-F_{h}\left[u\right]\left(t_{i}\right)<0. \end{array}\right.$$

The relationship between the bending moment $M_{a}\left(t\right)$, which is the input of the flexible arm, and the strain of the SMA wire, is considered linear. Based on Mohr’s theorem, the moment $M_{a}\left(t\right)$ is defined as Equation (11).

(11) $$M_{a}\left(t\right)=M_{a0}\cdot u^{*}\left(t\right),$$

where $M_{a0}$ is given by

(12) $$M_{a0}=\frac{3EI}{l_{1}^{2}}.$$

### 2.4. Modeling of the Flexible Arm

The equation of motion (13) is derived based on Euler–Bernoulli beam theory, which is a simplification of the linear isotropic theory of elasticity, which provides a means of calculating the load-carrying and deflection characteristics of beams. The formulation of the flexible arm refers to Reference [13].

(13) $$\rho S\frac{\partial^{2}y_{arm}\left(x,t\right)}{\partial t^{2}}+\frac{\partial^{2}}{\partial x^{2}}\left[EI\left(1+C_{i}\frac{\partial }{\partial t}\right)\frac{\partial^{2}y_{arm}\left(x,t\right)}{\partial x^{2}}\right]=\frac{\partial^{2}}{\partial x^{2}}[M_{a}\left(t\right)\delta \left(x-l_{1}\right)]$$

As shown in Figure 2, the elastic deformation of the flexible arm is modeled using the assumed modes approach; it is assumed that the flexural displacement of the flexible arm $y_{arm}\left(x,t\right)$ can be expressed in terms of the mode shapes as follows.

$$y_{arm}\left(x,t\right)=\sum_{i=1}^{\infty }\omega_{i}\left(x\right)f_{i}\left(t\right),$$

where $\omega_{i}\left(x\right)$ represents mode shape functions and $f_{i}\left(t\right)$ represents the generalized coordinates. Since the considered arm is completely fixed at one end and completely free at the other, there exists the following set of boundary conditions. The *i*th natural vibration mode function $\omega_{i}\left(x\right)$ can be expressed as:

$$\omega_{i}\left(x\right)=B_{i}\left[\left(\cosh\lambda_{i}l+\cos\lambda_{i}l\right)\left(\cosh\lambda_{i}x-\cos\lambda_{i}x\right)-\left(\sinh\lambda_{i}l-\sin\lambda_{i}l\right)\left(\sinh\lambda_{i}x-\sin\lambda_{i}x\right),\right]$$

where the flexural displacement of the flexible arm $y_{arm}\left(x,t\right)$ can be formulated by Equation (14).

(14) $$y_{arm}\left(x,t\right)=\sum_{i=1}^{\infty }\left[J_{i}\int_{0}^{t}e^{-\frac{\alpha_{i}}{2}\left(t-\tau \right)}\cdot \sin\frac{\beta_{i}}{2}\left(t-\tau \right)\cdot M_{a}\left(\tau \right)d\tau \right],$$

where $J_{i}$, $\alpha_{i}$, $\beta_{i}$, $k_{i}$ are obtained as below. $\omega_{i}$ is the *i*th natural vibration mode function.

$$\begin{array}{ccc} J_{i}=\frac{2\omega_{i}\left(x\right)}{\rho S\psi_{i}\beta_{i}}\omega_{i}^{\prime \prime }\left(l_{1}\right), & & \alpha_{i}=k_{i}^{2}C_{i}\\ \beta_{i}=\sqrt{4k_{i}^{2}-k_{i}^{4}C_{i}^{2}}, & & k_{i}=\sqrt{\frac{\lambda^{4}EI}{\rho S}}. \end{array}$$

## 3. Proposed Interactive Actuation Vibration Control

**Figure 4:**
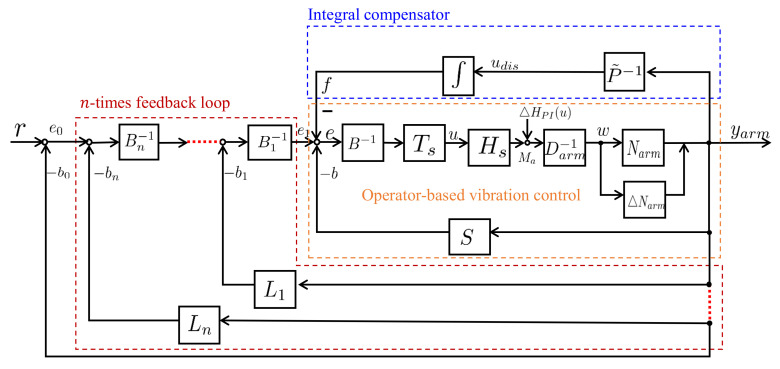
Framework of the proposed interactive actuation vibration control method.

According to the obtained models of the flexible arm, the framework of the proposed vibration control system is an interactive actuation integrated with a robust nonlinear vibration controller, a designed integral compensator, and a desired tracking *n*-times feedback loop as shown in Figure 4. In short, the robust stabilization and the desired tracking performance of nonlinear vibration control will be discussed in this section. These are divided into three subsections as follows.

- Nonlinear vibration controller guarantees the robust stabilization by using robust right coprime factorization;
- The designed integral compensator reduces the displacement vibration of the flexible arm;
- *n*-times feedback loop can obtain the desired tracking performance.

### 3.1. Robust Nonlinear Vibration Controller

The flexible arm’s first vibration mode is represented as an operator *P* of the nominal plant. The unconsidered vibration modes of the flexible arm are regarded as △P. $△N_{arm}$ is regarded as the uncertainties. The unconsidered hysteresis modes $△H_{PI}\left(u\right)\left(t\right)$ are regarded as a bounded disturbance. Then, the overall plant using right coprime factorization can be expressed as Equation (15):

(15) $$\left(P+△P)(M_{a})(t)=(N_{arm}+△N_{arm})D_{arm}^{-1}(M_{a})(t\right)$$

(16) $$(N_{arm}+△N_{arm})(w)(t)=(J_{1}+△J_{1})\;\;\int_{0}^{t}e^{-\frac{\alpha_{1}}{2}\left(t-\tau \right)}\sin\frac{\beta_{1}}{2}(t-\tau )\cdot w(\tau )d\tau$$

(17) $$D_{arm}(w)(t)=w(t).$$

The invertible operator ${\tilde{D}}^{-1}\left(u_{d}\right)\left(t\right)$ is defined by the thermal model and hysteresis model of the SMA actuator and the designed operator $D_{arm}^{-1}$ as Equation (18).

(18) $${\tilde{D}}^{-1}\left(u_{d}\right)\left(t\right)=D_{arm}^{-1}H_{s}T_{s}\left(u_{d}\right)\left(t\right).$$

When the operators *S* and *B* are designed to satisfy the Bezout identity, the nonlinear control system can be guaranteed BIBO stable.

(19) $$S\left(y_{arm}\right)\left(t\right)=K_{1}\frac{1}{A_{a1}*\beta_{1}}\left({\ddot{y}}_{arm}\left(t\right)+\alpha_{1}{\dot{y}}_{arm}\left(t\right)+0.25\left(\alpha_{1}^{2}+\beta_{1}^{2}\right)y_{arm}\left(t\right)\right)$$

(20) $$B\left(u_{d}\right)\left(t\right)=\left(1-K_{1}\right){\tilde{D}}^{-1}\left(u_{d}\right)\left(t\right),$$

where $K_{1}$ is a designed parameter, $y_{arm}$ is the output of the flexible arm’s displacement. The plant is said to be robust stable if the following robust stability conditions are satisfied in [39].

(21) $$∥\left[S\left(N_{arm}+\Delta N_{arm}\right)-SN_{arm}\right]{∥}_{Lip}<1.$$

### 3.2. A Designed Integral Compensator

The integral compensator is designed to mitigate the effects of the hysteresis property. The output signal f(t) of the integral compensator will be fed back to the vibration control loop.

(22) $$f(t)=K_{2}\int_{0}^{t}\frac{1}{A_{a1}*\beta_{1}}\left({\ddot{y}}_{arm}\left(\tau \right)+\alpha_{1}{\dot{y}}_{arm}\left(\tau \right)+0.25\left(\alpha_{1}^{2}+\beta_{1}^{2}\right)y_{arm}\left(\tau \right)\right)d\tau ,$$

where $K_{2}$ is a designed parameter.

Then, the designed parameters of $A_{a1}$, $\alpha_{1}$, $\beta_{1}$, $k_{1}$ are expressed by the following equations.

$$\begin{array}{ccc} A_{a1}=\frac{2\omega \left(x\right)}{\rho S\psi \beta_{m}}\omega^{\prime \prime }\left(l_{1}\right), & & \alpha_{i}=k_{1}^{2}C\\ \beta_{1}=\sqrt{4k_{1}^{2}-k_{1}^{4}C^{2}}, & & k_{i}=\sqrt{\frac{\lambda^{4}EI}{\rho S}}. \end{array}$$

### 3.3. Tracking Performance of n-Times Feedback Loop

The *n*-times feedback loop for obtaining the desired tracking performance is designed to satisfy the Bezout identity. The concept of the *n*-times feedback loop is cited in Reference [28]. The formulation of the *n*-times feedback loop is shown in the following.

(23) $$\begin{array}{cc} L_{1}\left(N_{arm}+▵N_{arm}\right)+B_{1}L & =M_{1}\\ & \vdots \\ L_{n}\left(N_{arm}+▵N_{arm}\right)+B_{n}M_{n-1} & =M_{n}. \end{array}$$

The designed controllers are shown as follows.

(24) $$L_{1}\left(y_{arm}\right)\left(t\right)=b\left(t\right)\frac{a_{0}}{\left(a_{0}+|b\left(t\right)|\right)}$$

(25) $$b(t)=k_{p}y_{arm}\left(t\right)+k_{d}{\dot{y}}_{arm}\left(t\right)$$

(26) $$L_{n}=k^{n}L_{1}$$

(27) $$B_{1}=\ldots =B_{n}=k_{c}<1,$$

where the designed parameters $a_{0}$, $k_{p}$, $k_{d}$, *k* (0 < *k* < 1), $k_{c}$ are 0.2, 1.0, 0.005, 0.9, and 0.95, respectively.

From Figure 4, assuming the system consists of only the operator-based vibration control loop and the *n*-times feedback loop without considering the integral compensator loop, the following equation can be obtained for the relationship between the output $y_{arm}$ and the reference input *r*. When each feedback loop of the designed *n*-times feedback loops satisfies the Bezout identity, the desired tracking performance can be obtained.

(28) $$\begin{array}{cc} y_{arm}\left(t\right) & =b_{0}\left(t\right)=r\left(t\right)-e_{0}\left(t\right)\\ & =r\left(t\right)-{\left(I+\left(N_{arm}+▵N_{arm}\right)M_{n}^{-1}\right)}^{-1}\left(r\left(t\right)-\left(N_{arm}+▵N_{arm}\right)M_{n}^{-1}{▵}_{0}\right), \end{array}$$

where ${▵}_{0}$ is expressed as $B_{n}\ldots B_{0}B{\tilde{D}}^{-1}▵H_{PI}$.

The conditions for tracking can be obtained as follows: If *n* → *∞*, conditions of $M_{n}$→ 0 can be derived from Equations (23)–(27),

(29) $${\left(I+\left(N_{arm}+▵N_{arm}\right)M_{n}^{-1}\right)}^{-1}\left(N_{arm}+▵N_{arm}\right)M_{n}^{-1}\to I$$

and $B_{n}\ldots B_{0}B{\tilde{D}}^{-1}$ → 0 can be obtained.

Therefore, the $▵H_{PI}$ can be made arbitrarily small.

Then, $\left(y_{arm}\left(t\right)-r\left(t\right)\right)$ can be made arbitrarily small. The desired tracking performance can be obtained.

## 4. Results and Discussion

In the results of simulation case studies, the effectiveness of the proposed vibration control method using interactive actuation was verified by simulation results. As a partial achievement of research on the same topic, it is necessary to restate a few important conclusions. The simulation results showed that the vibration control with the designed integral compensator can reduce the displacement vibration of the flexible arm more than one without this compensator. The desired tracking performance can be obtained by increasing the *n*-times feedback loop. In a comparison of a traditional PD controller and a single-sided SMA actuator, the simulation results proved that the proposed nonlinear vibration control using a double-sided SMA actuator is more effective with a shorter duration and less input power. The further experimental system design is to demonstrate the effectiveness of the proposed nonlinear vibration control using interactive SMA actuation.

### 4.1. Experiment System

**Figure 5:**
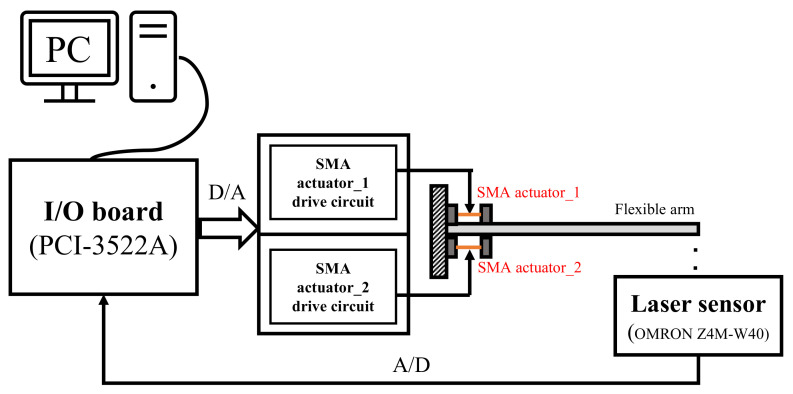
Schematic of the experimental setup.

The schematic diagram of the experimental system is presented in Figure 5. This experimental system consists of a flexible arm with an interactive SMA actuator, a laser sensor, and a PC-based control board. Detailed information was introduced in Section 2. The input signal generated by the PC-based controller excited the SMA actuator’s drive circuit through the interface I/O board. The bending moment generated by the interactive SMA actuator acted on the flexible arm to suppress the displacement vibration. The laser sensor measured this displacement vibration at the bottom of the flexible arm’s end side.

The parameters in experimental cases are listed in Table 2. Input electric power $u_{d}$, as in Equation (30), which was the same as in the simulation, was added to make the flexible arm vibrate during the first 5 s. The input electric current was limited to 2.16 amps for safe experimental operation. After 5 s, it vibrated freely and the PC-based controller generated the control signal to suppress the vibration of the flexible arm through the interactive SMA actuation. The sampling time of the experiment was 0.02 s.

(30) $$u_{d}\left(t\right)=0.7\sin\left(1.46\pi t\right).$$

### 4.2. Experimental Results

The experimental tests were conducted for three cases.

**Figure 6:**
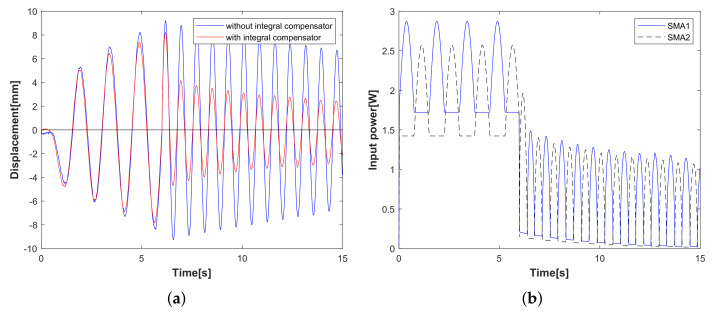
Experimental case 1: vibration control with/without integral compensator. (**a**) Displacement of the flexible arm. (**b**) Input power of SMA actuator.

**Figure 7:**
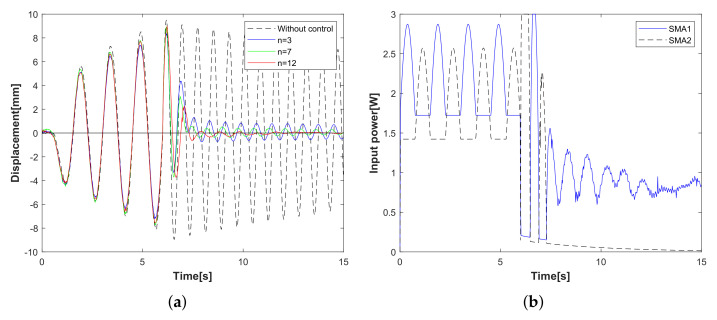
Experimental case 2: the tracking performance of *n*-times feedback loop. (**a**) Displacement of the flexible arm (*n* = 3, 7, 12). (**b**) Input power of SMA actuator (*n* = 12).

**Figure 8:**
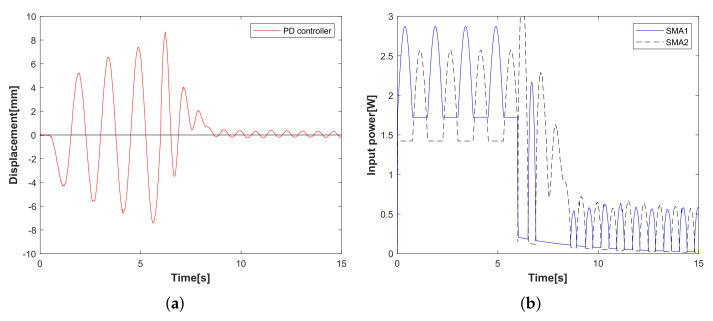
Experimental case 3: Vibration control with conventional PD controller. (**a**) Displacement of the flexible arm. (**b**) Input power of SMA actuator.

- Experimental case 1: operator-based vibration controller with/without the designed integral compensator. In Figure 6, the displacement of the flexible arm decreases after 5 s; the result is verified that the operator-based vibration controller provides robust stability. Compared with the displacement of the red line and the blue line, it is clear that the designed integral compensator as a red line can reduce the displacement vibration of the flexible arm shown in Figure 6a, and the input power to the SMA actuator is shown in Figure 6b. From experimental case 1, when the vibration controller is integrated with the designed integral compensator, the vibration displacement of the flexible arm is reduced by more than that without the integral compensator.
- Experimental case 2: the tracking performance of the *n*-times feedback loop. In Figure 7, the tracking performance of the *n*-times feedback loop with *n* = 3, 7, 12 is presented. The experimental results can confirm that the output performance of the *n*-times feedback loop is obviously stable within 8 s in Figure 7a and with lower input power (*n* = 12) in Figure 7b. While increasing the number of *n*, the vibration suppression has a fast response with less waveform, and the desired tracking performance is improved more effectively. From experimental case 2, compared to free vibration control, the displacement of the flexible arm is shown as a red line for the 12-times feedback loop and a green line for 7-times and a blue line for the 3-times feedback loop. The tracking performance is optimized by increasing the number of 3, 7, 12 times feedback loops.
- Experimental case 3: vibration attenuation of the conventional PD controller. The designed parameters of PD controllers $K_{P}$ and $K_{D}$ are 600 and 150, respectively. In Figure 8, a conventional PD controller of the flexible arm is compared. Vibration attenuation of PD controller becomes stable beyond the 7th vibration waveforms in Figure 8a, while the input power in Figure 8b is higher than that of the proposed method in Figure 7b. From the experimental case 3, the conventional PD controller stabilizes the vibration displacement after the 7th vibration waveform, while the vibration displacement approaches zero after the 4th vibration waveform using the proposed vibration control method, which is proved to be faster and more effective in controlling the flexible arm’s vibration. This paper focuses on the qualitative analysis of the vibration control speed and input power magnitude.

This paper focuses on the evaluation of vibration control speed and input power magnitude. Future research will consider a quantitative analysis. From the above experimental results, the effectiveness of the proposed vibration control method is demonstrated. The experimental results are also in good agreement with the simulation results.

## 5. Conclusions

This paper developed an experimental system of nonlinear vibration control using an interactive actuation for a flexible arm. The operator-based vibration controller, the designed integral compensator, and the *n*-times feedback loop of operators were all incorporated into the vibration suppression approach. In the experiment results, the nonlinear vibration controller with an integral compensator ensured the robust stability, and reduce the displacement, of the flexible arm. The flexible arm’s tracking performance may be improved even further by increasing the *n*-times feedback loop. Three experimental strategies demonstrated that the proposed interactive SMA actuator method has more effective vibration suppression and a better tracking performance than the conventional PD controller. To analyze the effect of feedback loops on tracking performance, the number of feedback loops was set to three values by trial and error to find a desirable result. The limitation of this study was that it did not consider how to find the optimal number of feedback loops. The problem of the optimal number of feedback loops needs further study. The focus of this work was on the evaluation of input power magnitude and vibration control speed. A quantitative analysis will be considered in future studies. Future work will consider vibration control integrated with fault tolerance capacity even if the interactive SMA actuator is faulty.

## Figures and Tables

**Table 1:** Physical parameters of the flexible arm with SMA wire.

Description	Symbol [Unit]	Value
Length of SMA wire	$l_{1}\left[m\right]$	0.1
Diameter of SMA wire	*d* [m]	1 × 10^−4^
Resistance of SMA wire	*R* [Ω]	13.5
Heat transfer coefficient	$h_{c}\left[W/\left(m^{2\circ }C\right)\right]$	689
Surface area of SMA wire	$A_{c}\left[m^{2}\right]$	3.14 × 10^−7^
Mass of SMA wire	*m* [kg]	5 × 10^−6^
Length of the flexible arm	*l* [m]	0.8
Specific heat	$c_{p}\left[J/\left({\mathrm{kg}}^{\circ }C\right)\right]$	7349
Density	*ρ* [kg/m^3^]	2700
Cross-sectional area	*S* [m^2^]	10 × 10^−6^
Young’s modulus	*E* [N/m^2^]	6.9 × 10^10^
Area moment of inertia	*I* [m^4^]	1.67 × 10^−12^
1st order damping mode)	*C*_1_ [s]	0.0015
Ambient temperature	*T_a_* [°C]	20

**Table 2:** Experimental parameters.

Parameters	Symbol [Unit]	Value
Sampling time	[s]	0.02
Experiment time	[s]	15
Ambient temperature	*T_a_* [°C]	20
Designed parameter	*K*_1_ [−]	0.02
Designed parameter	*K*_2_ [−]	0.02

## Data Availability

Data are not publicly available due to privacy considerations.
